# How RIVM is responding to the challenge of protecting human health from the effects of climate change – now and in the future

**DOI:** 10.1093/eurpub/ckac129.225

**Published:** 2022-10-25

**Authors:** L Hall, J Zwartkruis, G Velders

**Affiliations:** Environment and Safety Division, RIVM, Bilthoven, Netherlands

## Abstract

In 2020 the Dutch National Institute for Public Health and the Environment (RIVM) published its vision on climate change and health. This vision reflects the position of RIVM in society and highlights the transdisciplinary and collaborative nature of our work on this topic. We define this societal challenge as: protecting and promoting the health of people throughout society with regard to climate change and climate measures. The purpose of our work on this challenge is to support all levels of Dutch government with policy development and implementation and to forge links with both national and international organisations to further knowledge and expertise.

The focus of our work, in collaboration with the aforementioned actors, is as follows:

1. Knowledge translation of the health impacts of climate change for the public and government.

2. Developing instruments to promote health in relation to climate change and mitigation and adaptation measures.

3. Implementation research on solutions to mitigate and adapt to climate change.

4. Strengthening national and international cooperation to enhance synergies in our work.

In the workshop we will present examples of our work:

• Quantifying the health impacts of climate change, now and in the future under different global warming scenarios.

• Developing a set of indicators to monitor the health impacts of climate change and mitigation and adaptation plans and actions.

• Collating and disseminating easily digestible information for public health services on evidence-based, effective adaptation measures.

• Developing tools and instruments to ensure health benefits and limit unintended consequences of adaptation measures, such as green and blue spaces.

• Prioritising research on climate change and health through a research agenda.

Workshop participants will be encouraged to provide feedback to inform RIVM's future work and that of other national public health institutes, and to ensure we are fully supporting our stakeholders.

